# PHRF1 promotes genome integrity by modulating non-homologous end-joining

**DOI:** 10.1038/cddis.2015.81

**Published:** 2015-04-09

**Authors:** C-F Chang, P-C Chu, P-Y Wu, M-Y Yu, J-Y Lee, M-D Tsai, M-S Chang

**Affiliations:** 1 Institute of Biological Chemistry, Academia Sinica, Taipei 11529, Taiwan; 2 Institute of Biochemical Sciences, College of Life Science, National Taiwan University, Taipei 11529, Taiwan

## Abstract

Methylated histone readers are critical for chromatin dynamics, transcription, and DNA repair. Human PHRF1 contains a plant homeodomain (PHD) that recognizes methylated histones and a RING domain, which ubiquitinates substrates. A recent study reveals that PHRF1 is a tumor suppressor that promotes TGF-*β* cytostatic signaling through TGIF ubiquitination. Also, PHRF1 is a putative phosphorylation substrate of ataxia telangiectasia-mutated/ataxia telangiectasia and Rad3-related kinases; however, the role of PHRF1 in DNA damage response is unclear. Here we report a novel function of PHRF1 in modulating non-homologous end-joining (NHEJ). PHRF1 quickly localizes to DNA damage lesions upon genotoxic insults. Ablation of PHRF1 decreases the efficiency of plasmid-based end-joining, whereas PHRF1 overexpression leads to an elevated NHEJ in H1299 reporter cells. Immunoprecipitation and peptide pull-down assays verify that PHRF1 constitutively binds to di- and trimethylated histone H3 lysine 36 (H3K36) (H3K36me2 and H3K36me3) *via* its PHD domain. Substitution of S^915^DT^917^E to ADAE in PHRF1 decreases its affinity for NBS1. Both PHD domain and SDTE motif are required for its NHEJ-promoting activity. Furthermore, PHRF1 mediates PARP1 polyubiquitination for proteasomal degradation. These results suggest that PHRF1 may combine with H3K36 methylation and NBS1 to promote NHEJ and stabilize genomic integrity upon DNA damage insults.

Unrepaired double-strand breaks (DSBs) can lead to oncogenic transformations or cell death. In higher eukaryotes, DSBs are mainly repaired by homologous recombination (HR; error-free repair) and non-homologous end-joining (NHEJ; error-prone repair).^1, 2^ It has been suggested that DSB response mainly relies on the *γ*-H2AX-MDC1-RNF8-RNF168 axis, but diverges to BRCA1 for HR and 53BP1 for NHEJ.^1, 2^ The definitive step for HR or NHEJ is dependent on Rap1-interacting factor 1 (Rif1). In the absence of Rif1, DNA ends undergo BRCA1-dependent resection to facilitate HR. In the G1 phase, Rif1 in association with the phosphorylated 53BP1 protects DNA end resection mediated by BRCA1-CtIP and consequently promotes NHEJ.^3, 4, 5, 6^ Classical NHEJ mainly occurs in the G1 phase. Ku70/Ku80 heterodimer binds to DNA ends and recruits DNA-PKcs, Artemis, and Pol *μ*/*λ* to the repair site, resulting in end processing followed by ligase IV, XRCC4, and XLF-mediated ligation.^7^ In the absence of Ku proteins, Mre11-RAD50-NBS1 (MRN) in conjunction with CtIP facilitate alternative NHEJ (alt-NHEJ) using short microhomologies^8^. Furthermore, MRN recognizes DSBs and promotes HR during the S/G2 phase.^8^

Histone methylation has key roles in chromatin remodeling and regulation of gene transcription, DNA replication, genome stability, and apoptosis.^9, 10^ Histone lysine residues can be monomethylated (me1), dimethylated (me2), or trimethylated (me3) on particular histone methylation sites, such as histone H3 lysine 4 (H3K4), H3K9, H3K27, H3K36, H3K79, and H4K20.^9, 10^ Methylated histones are recognized by readers containing specific domains, such as plant homeodomain (PHD), WD40 repeat, chromodomain, and Tudor domain.^11, 12, 13, 14, 15, 16^ Readers that recognize methylated histones could recruit other chromatin remodeling enzymes to render *cis*- or *trans*-modifications on histones, thus regulating chromatin dynamics and controlling cellular functions.

Several studies have reported the connection between histone methylation and NHEJ. Notably, 53BP1 recruitment to DNA lesions is through the binding of its tudor domain with increased dimethylated H4K20 (H4K20me2) at local DNA damage sites during NHEJ.^17, 18^ The methylation of H4K20me2 by histone methyltransferase MMSET in mammals is critical for the recruitment of 53BP1 to DSBs.^19^ However, this scenario has been argued by other groups because they could not detect any defects in 53BP1 recruitment in MMSET-deficient cells. Instead, H3K79me2 is required for 53BP1 foci formation during the G1/G2 phases while the level of H4K20me2 is low.^20^ Recently, H3K36me2 has been demonstrated to be involved in NHEJ pathway. Locally increased H3K36me2 improves the association of Ku70 and NBS1 and promotes DSB repair by NHEJ, possibly mediated by an unidentified histone reader protein.^21^

Ataxia telangiectasia-mutated (ATM) and ataxia telangiectasia and Rad3-related (ATR) kinases are important signaling kinases in DNA damage response.^1, 2^ Using anti-pSQ/pTQ antibody to perform a large-scale identification of ATM and ATR-phosphorylated targets, PHRF1 is found to be phosphorylated on S925 and S1389 in response to ionizing irradiation.^22^ The primary structure shows that PHRF1 contains one RING domain (amino acid (a.a.) 106–150) for E3 ligase activity, one PHD domain (a.a. 185–233) for association with methylated histones, one putative CK2 phosphorylation motif (SDTE, a.a. 915–918), and seven SQ/TQ motifs mainly located on its C terminus. Very recently, PHRF1 is demonstrated as a tumor suppressor responsible for the ubiquitination of TGIF to release cPML and promote the TGF-*β* cytostatic program.^23^ In an effort to illustrate the function of PHRF1 in DNA damage response, we analyzed cellular responses to genotoxic treatments in PHRF1-knockdown and -overexpressing cells. Our results suggest that PHRF1 associates with methylated H3K36 and interacts with NBS1 to promote NHEJ in response to genotoxic stress.

## Results

### PHRF1 binds to DNA lesions upon genotoxic stress

**Figure 1:**
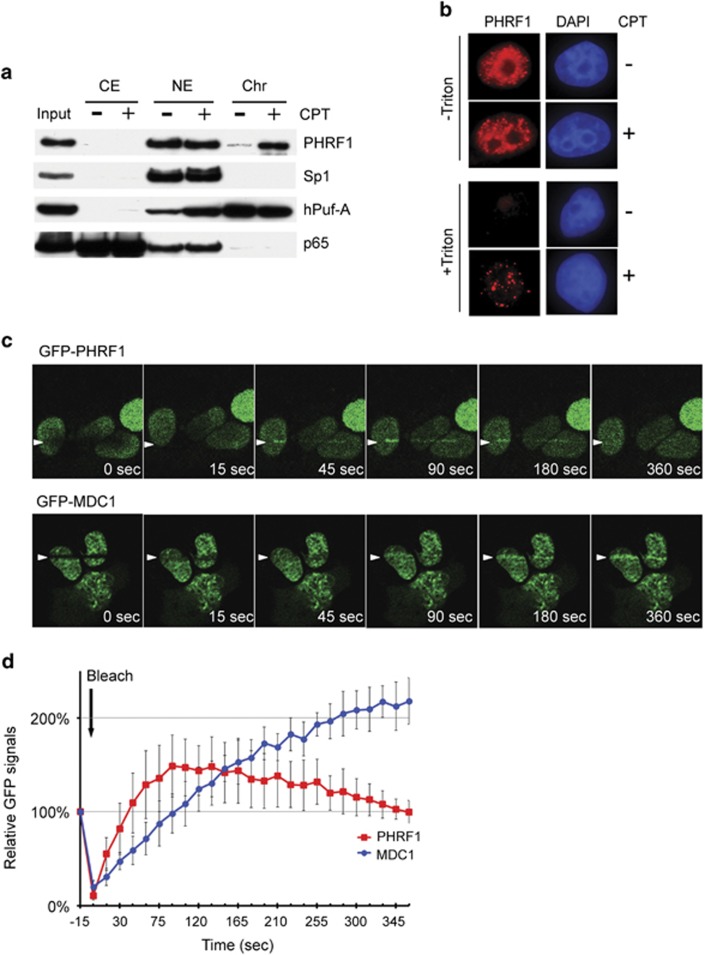
PHRF1 associates with chromatin after genotoxic stress. (**a**) Subcellular fractionation of HeLa cell extracts was prepared for immunoblot analysis. P65 is a subunit of nuclear factor-*κ*B (NF-*κ*B) as a CE marker. Sp1 is an NE marker. hPuf-A, a nucleolar protein moving from chromatin to nuclear extracts upon genotoxic stress. (**b**) HeLa cells were pre-extracted by cytoskeleton extraction buffer containing 0.5% Triton X-100 and fixed in paraformaldehyde for immunofluorescence (bar=10 *μ*m). (**c**) Dynamic recruitment of GFP-PHRF1 to DNA damage sites generated by laser microirradiation in U2OS cells. GFP-MDC1 was a control. (**d**) Kinetics of the recruitment of GFP-PHRF1 and GFP-MDC1 to DNA damage sites were analyzed from 10 microirradiated cells in each experiment. The highest GFP intensity was calculated as 100% and data were presented as mean±S.D. CE, cytosolic extracts; Chr, chromatin-enriched fraction; GFP, green fluorescent protein; NE, nuclear extracts; WCE, whole-cell extracts

As PHRF1 is an ATM/ATR-phosphorylated substrate, we set out to examine whether PHRF1 associated with chromatin in response to genotoxic stress. Subcellular fractionation showed that PHRF1 was mainly present in soluble nuclear extracts without camptothecin (CPT, a topoisomerase I inhibitor) treatment. In contrast, the amount of PHRF1 was elevated in the chromatin fraction after CPT treatment (Figure 1a). To visualize the chromatin association of PHRF1, HeLa cells were exposed to CPT and then pre-extracted with 0.5% Triton X-100. Triton-extracted cells also showed an increased chromatin-associated PHRF1 after CPT exposure (Figure 1b).

To further confirm that PHRF1 was recruited to DNA damage sites, we monitored the *in vivo* recruitment of GFP-PHRF1 in response to laser-induced DNA lesions. Interestingly, live-cell imaging revealed a fast accumulation of PHRF1 at the irradiated sites followed by a rapid decrease reaching to the basal fluorescence level, with a peaking at 90 s and lasting to 5 min after laser irradiation. By contrast, GFP-MDC1, a key mediator in DNA damage response that recognizes *γ*-H2AX signal, exhibits a much slower recruitment than that of PHRF1 under the same laser irradiation, appearing after 90 s and remaining accumulated to the end of experiment (Figure 1c). Quantitatively, the protein fluorescence at laser-irradiation site shows distinct recruitment kinetics of PHRF1 and MDC1, in which PHRF1 appeared at DNA damage sites more quickly but transiently compared with MDC1 (Figure 1d). These results likely suggest that the recruitment of PHRF1 is a very early event of DNA damage response. In agreement, PHRF1 did not localize to MDC1-associated foci at 2 h after laser irradiation in GFP-MDC1 cells (Supplementary Figure S1) nor colocalize with any of *γ*-H2AX, RNF8 or BRCA1 following 3 h CPT treatment in HeLa cells (Supplementary Figure S1). This transient recruitment behavior possibly resulted in the exclusion of PHRF1 from the *γ*-H2AX- and MDC1-associated nuclear foci upon longer postincubation time.

### PHRF1 affects cell viability in response to genotoxic insults

**Figure 2:**
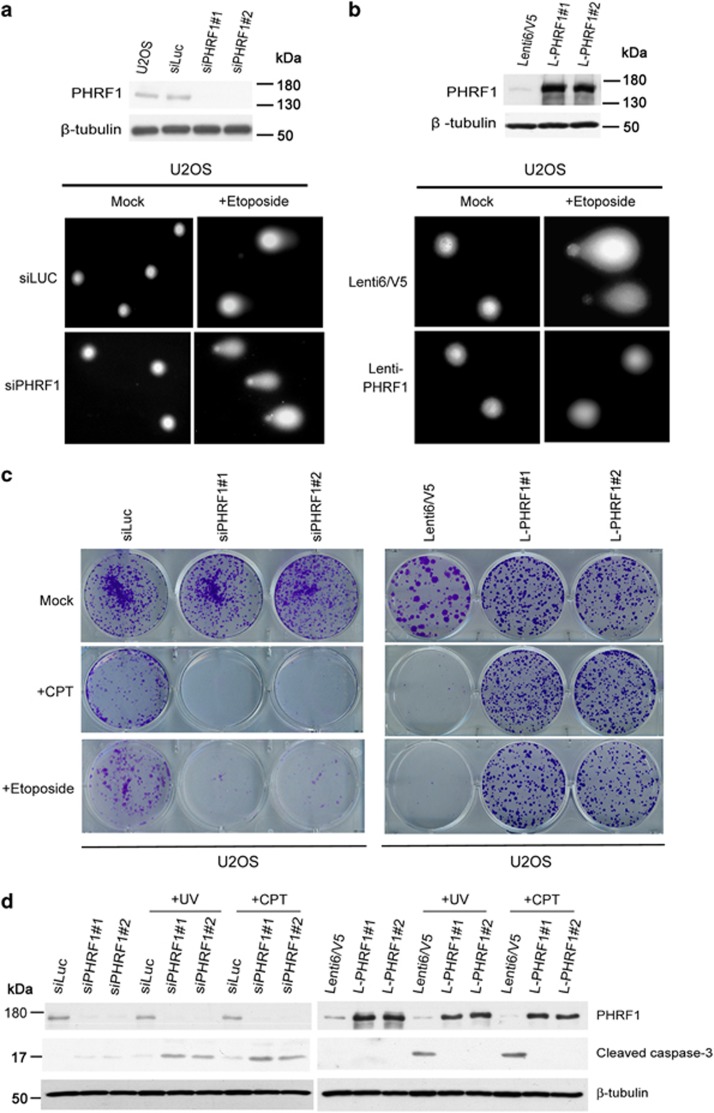
PHRF1 protects cells from genotoxic insults. (**a**) PHRF1-depleted U2OS cells were exposed to etoposide for 30 min and then collected for the comet assay. (**b**) Control and PHRF1-overexpressing U2OS cells were exposed to etoposide for 1 h and then collected for the comet assay. (**c**) PHRF1-depleted U2OS cells were exposed to CPT (2 *μ*M) for 24 h or etoposide (10 *μ*M) for 1 h and PHRF1-overexpressing cells were treated with CPT (5 *μ*M) for 24 h or etoposide (25 *μ*M) for 1 h for clonogenic assays. Colonies were allowed to form for 10 days and stained with crystal violet. (**d**) PHRF1-depleted and -overexpressing cells were treated with CPT (10 *μ*M) and UV light (20 J/m^2^). Total cell extracts were collected after 2 h and immunoblotted with anticleaved caspase-3 antibody to examine apoptosis

To investigate whether PHRF1 protects the chromosome integrity, we analyzed chromosome lesions using the comet assay. The majority of PHRF1-depleted U2OS cells exposed to etoposide exhibited damaged and fragmented DNA with distinct tails. By contrast, most of the control cells did not have similar DNA tails (Figure 2a). Conversely, PHRF1-overexpressing U2OS cells migrated with normal patterns, but ~80% of control U2OS cells had fragmented DNA tails (Figure 2b). We examined whether PHRF1 rendered cells resistance to genotoxic insults by clonogenic assay. PHRF1-depleted U2OS cells barely survived after CPT or etoposide treatments. By contrast, PHRF1-overexpressing U2OS cells had much higher viability than control cells after genotoxic treatments (Figure 2c). Similar results for comet and clonogenic assays were revealed in PHRF1-depleted and -overexpressing HeLa cells and also in breast cancer MDA-MB-231 cells (Supplementary Figure S2). We measured the effects of PHRF1 on cell cycle progression by flow cytometry. The addition of CPT in PHRF1-knockdown and -overexpressing cells did not result in accumulated phases in cell cycle progression. Instead, an increased sub-G1 population in PHRF1-depleted U2OS cells and a reduced sub-G1 population in PHRF1-overexpressing U2OS cells exposed to CPT suggested that PHRF1 is required for cell viability upon genotoxic insults. Again, similar results were found in PHRF1-depleted HeLa and MDA-MB-231 cells (Supplementary Figure S3). We further analyzed the expression of cleaved caspase-3 to confirm whether PHRF1 affected cell viability via apoptosis. The degraded and activated product of caspase-3 was increased in PHRF1-depleted cells, whereas this apoptotic marker was decreased in PHRF1-overexpressing cells after UV irradiation or CPT exposure (Figure 2d). Collectively, these data suggested that PHRF1 modulates apoptosis upon genotoxic stress.

### PHRF1 does not affect DNA damage signaling response

To investigate whether PHRF1 affects DNA damage response, we examined the phosphorylation levels of RPA2 (a single-strand binding protein) and *γ*-H2AX (a marker for DSB) in the presence or absence of PHRF1. There was no significant change in phosphorylated RPA2, *γ*-H2AX, Chk1 at S345, and Chk2 at T68 in PHRF1-depleted cells. Total amounts of RNF8 and RNF168 were also unchanged (Supplementary Figure S4). Additionally, the foci formation of RNF8, 53BP1, and BRCA1 was not affected in PHRF1-depleted and -overexpressing cells (Supplementary Figure S4), suggesting that PHRF1 may not impair DNA damage response.

We speculated that HR repair might be modulated by PHRF1. Nonetheless, the depletion and overexpression of PHRF1 did not alter the formation of rad51 foci (Supplementary Figure S5) and the proportion of GFP-positive cells in HR reporter DR-GFP cells was slightly changed in PHRF1-depleted and -overexpressing cells (Supplementary Figure S5), indicating that PHRF1 might not affect the efficiency of HR.

### PHRF1 associates with histone H3K36me2 and H3K36me3

**Figure 3:**
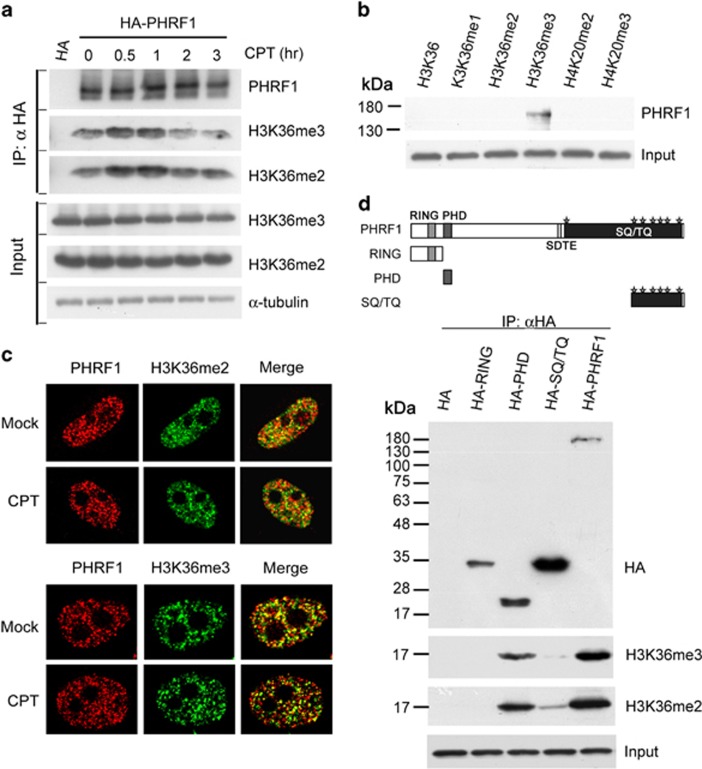
PHRF1 associates with H3K36me2 and H3K36me3. (**a**) Empty hemagglutinin (HA) vector and HA-PHRF1 were transfected into HEK293T cells for 48 h and cell extracts were immunoprecipitated (IP) with anti-HA agarose. Immunoblotting analysis was conducted using indicated anti-methyl histone antibodies. (**b**) Synthetic biotin-labeled H3K36-methylated peptides were incubated with HeLa cell extracts. Immunoblotting analysis was carried out using anti-PHRF1 antibody. (**c**) HeLa cells were simultaneously labeled with a mixture of anti-PHRF1 monoclonal antibody (mAb) (red) and anti-H3K36me2 or anti-H3K36me3 polyclonal antibodies (green). (**d**) HA-RING, HA-PHD, HA-SQ/TQ motif, and HA-PHRF1 were transfected into HEK293T cells and immunoprecipitated with anti-HA agarose. Immunoblotting was carried out using indicated antibodies. Note that there are seven SQ/TQ motifs in PHRF1

Immunoprecipitation and immunoblotting analyses showed that PHRF1 is associated with H3K36me2 and H3K36me3. A time-course study on immunoprecipitates of PHRF1 showed that the association of PHRF1 with H3K36me2/3 was increased at 0.5 and 1 h but declined at 1 h after CPT exposure. (Figure 3a). It has been suggested that an undefined reader protein might recognize H3K36me2 and H3K36me3 near DSBs to promote the binding of MRN complex and Ku70 for NHEJ repair.^24^ It is most likely that the elevated association of PHRF1 with H3K36me2/3 is due to locally increased H3K36me2/3 upon DNA damage, which has been suggested in *γ*-irradiation.^24^ Endogenous PHRF1 was specifically pulled down by biotin-labeled H3K36me3 peptide *in vitro*, but not by other peptides (Figure 3b). Interestingly, HA-PHRF1 purified from HEK293T cells could associate with biotin-labeled H3K36me2 and H3K36me3 (data not shown), suggesting that PHRF1 may interact with H3K36me2 under certain circumstances. When HeLa cells were simultaneously labeled with anti-PHRF1, anti-H3K36me2, and anti-H3K36me3 antibodies, the image showed large amount of overlapping areas in the nucleus (Figure 3c). By contrast, PHRF1 was not immunoprecipitated by other methylated histones including H3K4, H3K9, H3K27, H4K20 and acetylated histones (Supplementary Figure S6). Additionally, PHRF1 did not colocalize with these methylated histones as revealed by immunofluorescence analysis (Supplementary Figure S6).

Subsequently, to determine which region of PHRF1 is responsible for its association with H3K36me3, HA-tagged PHRF1 and its deletion mutants lacking RING, PHD, and SQ/TQ motifs were transfected into HEK293T cells. Immunoprecipitation by anti-HA agarose followed by immunoblotting analysis showed that PHD domain, but not RING or SQ/TQ motifs, is required for association with H3K36me2 and H3K36me3 (Figure 3d). Collectively, PHRF1 is a methylated histone reader to recognize H3K36me2 and H3K36me3 via its PHD domain.

### PHRF1 affects NHEJ *in vivo*

**Figure 4:**
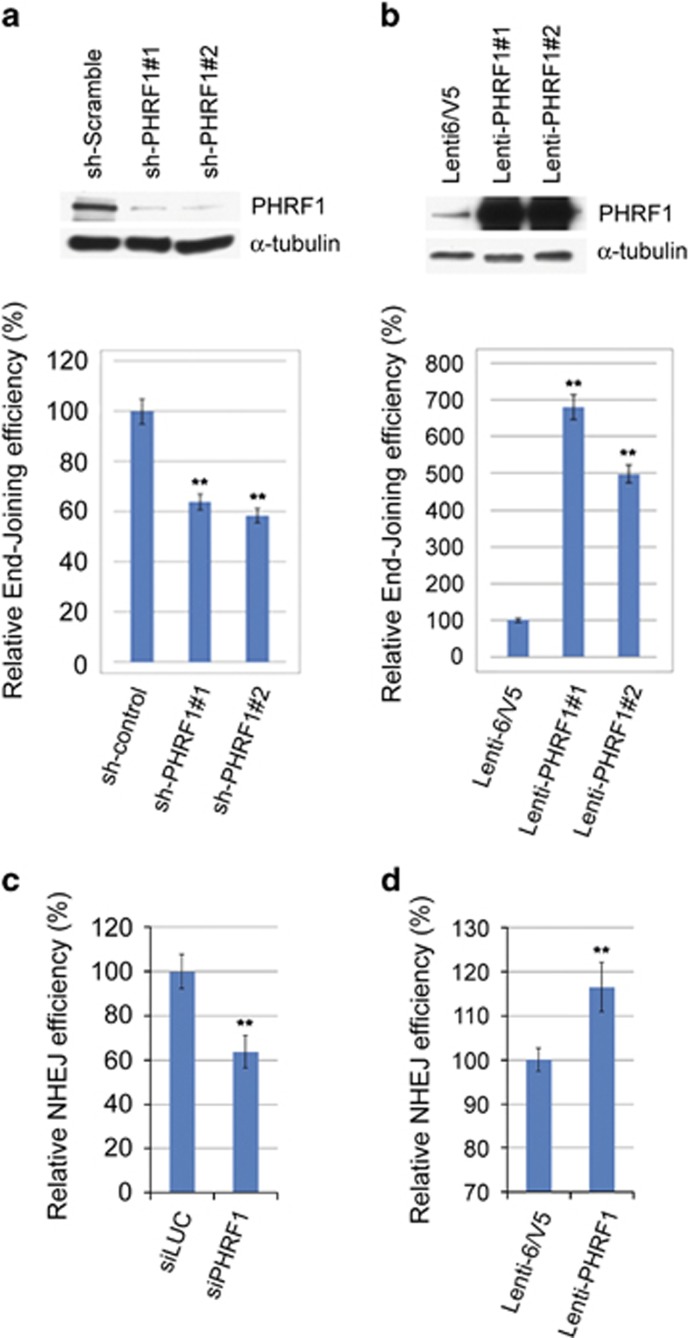
PHRF1 modulates NHEJ. *Hind*III-linearized pGL3 plasmid containing the *luciferase* gene was transfected into PHRF1-depleted HEK293T cells (**a**) and PHRF1-overexpressing U2OS cells (**b**), respectively. *In vivo* end-joining efficiency was measured by luciferase activity and normalized with *β*-gal expression. The data shown were mean±S.D. of three independent experiments. NHEJ reporter H1299dA3-1 no. 2 cells were transfected with PHRF1 siRNAs (**c**) or HA-PHRF1 (**d**) for 24 h and then transfected with I-SceI expression plasmid for another 48 h. The proportion of enhanced green fluorescent protein (EGFP)-positive cells was determined by flow cytometry at 48 h after I-SceI transfection. Each experiment represents the mean±S.D. of three independent experiments. *P<0.05 and **P<0.01 compared with the control. HA, hemagglutinin; IP, immunoprecipitation

To address whether PHRF1 is involved in linking methylated histone to NHEJ, a *Hind*III-linearized pGL3 plasmid containing the *luciferase* gene was transfected into PHRF1-depleted HEK293T cells and PHRF1-overexpressing U2OS cells, respectively. The results showed that end-joining activity was significantly reduced in PHRF1-knockdown cells, but increased in PHRF1-overexpressing cells compared with control cells (Figures 4a and b). Additionally, using a GFP-based NHEJ reporter H1299 human lung cancer cell harboring IRES-TK-EGFP DNA integrated in the genome,^25^ the proportion of EGFP-positive cells was decreased in PHRF1-knockdown cells, but increased in PHRF1-overexpressing cells after I-SceI transfection (Figures 4c and d). These data further support that PHRF1 modulates NHEJ *in vivo*.

### SDTE motif is required for the association of PHRF1 with NBS1

**Figure 5:**
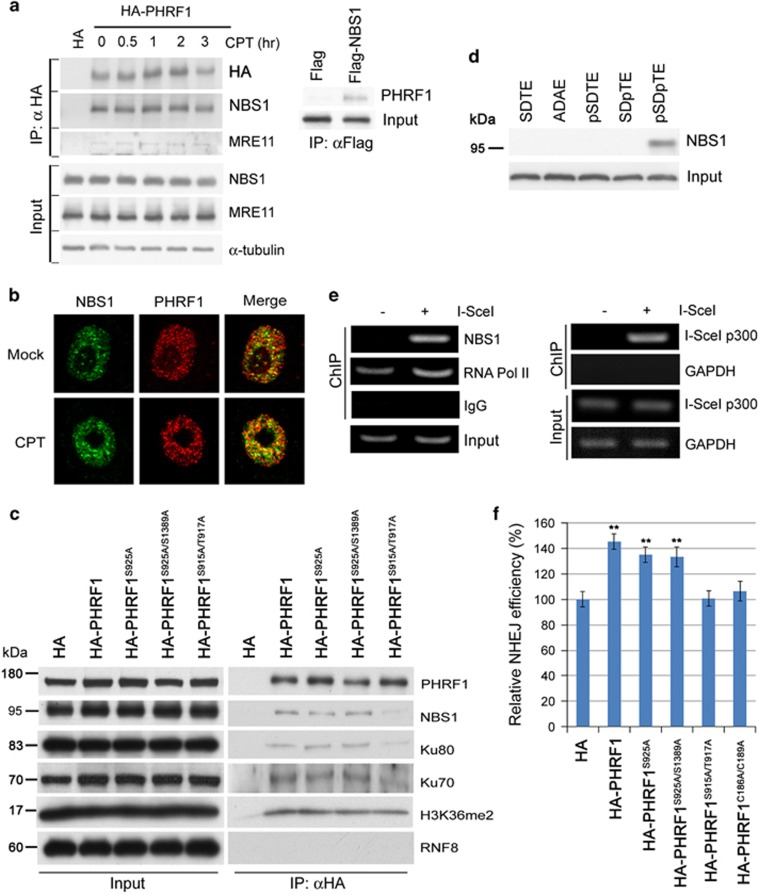
PHRF1 interacts with NBS1 via SDTE motif. (**a**) HeLa cells were transfected with empty FLAG vector and FLAG-NBS1 construct. Cell extracts were immunoprecipitated (IP) with anti-FLAG agarose and immunoblotted (IB) with anti-PHRF1 antibody. Reciprocal transfection with empty hemagglutinin (HA) vector and HA-PHRF1 was conducted. Input, the same amount of cell extracts for immunoprecipitation. (**b**) HeLa cells were exposed to CPT (10 *μ*M) for 2 h and then simultaneously labeled with a mixture of anti-PHRF1 mAb (red) and anti-NBS1 polyclonal antibody (green). (**c**) Cell extracts harvested from empty HA vector-, HA-PHRF1-, HA-PHRF1^S925A^-, HA-PHRF1^S925A/S1389A^- and HA-PHRF1^S915A/T917A^-transfected HEK293T cells were IP with anti-HA agarose and IB with indicated antibodies. (**d**) Synthetic biotin-labeled S^915^DT^917^E, A^915^DA^917^E, pS^915^DTE, SDpT^917^E, and pS^915^DpT^917^E peptides (a.a. residues 908–924 of PHRF1) were incubated with HeLa cell extracts. IB analysis was carried out using anti-PHRF1 antibody. (**e**) ChIP was performed by anti-NBS1 antibody to detect the region of 0.3 kb downstream of I-SceI site. Control IgG for ChIP and glyceraldehyde 3-phosphate dehydrogenase (GAPDH) for PCR were as controls. (**f**) Empty HA vector, HA-PHRF1, HA-PHRF1^S925A^, HA-PHRF1^S925A/S1389A^, PHRF1^S915A/T917A^, and HA-PHRF1^C186A/C189A^ constructs were transfected into NHEJ reporter H1299 cells. The proportion of enhanced green fluorescent protein (EGFP)-positive cells was determined by flow cytometry at 48 h after I-SceI transfection. Each experiment represented the mean±S.D. of three independent experiments. *P<0.05 and **P<0.01 compared with the control

SDTE motif is a conserved CK2 phosphorylation site and may interact with the FHA domain of NBS1.^26, 27, 28^ As PHRF1 contains one SDTE motif, we set out to investigate whether PHRF1 modulates NHEJ activity by interacting with NBS1. Immunoprecipitation followed by immunoblotting analysis showed that PHRF1 and NBS1 were in the same immunocomplex. PHRF1 predominantly associated with NBS1 and little amount of Mre11 was in the precipitates of PHRF1 (Figure 5a). Consistent with this, coimmunostaining studies of HeLa cells treated with 10 *μ*M CPT for 2 h revealed a partial colocalization of PHRF1 with NBS1 (Figure 5b). To elucidate the mechanistic interaction between PHRF1 and NBS1, we substituted SDTE to S915A/T917A in PHRF1 (PHRF1^S915A/S917A^) and S925/S1389 with alanine in PHRF1 (PHRF1^S925A/S1389A^) and then transfected these mutants into HEK293T cells. All substitution mutants of PHRF1 were listed in Supplementary Figure S7. As anticipated, a mutant PHRF1 protein in which S915 and T917 were substituted with alanine showed significantly reduced association with NBS1, whereas S925A and S925A/S1389A mutants did not alter the formation of this complex (Figure 5c). Furthermore, using synthetic peptides labeled with biotin, we found that only pS^915^DpT^917^E double-phosphorylated peptide, but not single phosphorylated or unphosphorylated peptides, could pull down endogenous NBS1 (Figure 5d).

Furthermore, DSBs were generated by I-SceI digestion in NHEJ reporter H1299 cells and chromatin immunoprecipitation (ChIP) by anti-NBS antibody was conducted. PCR analysis to detect a 0.3 kb region downstream of the second I-SceI site showed an enrichment of NBS1 and PHRF1 in this region after I-SceI transfection (Figure 5e), supporting the notion that PHRF1 and NBS1 were recruited to the sites of DSBs *in vivo*. Finally, SDTE mutant PHRF1^S915A/S917A^ and PHD mutant PHRF1^C186A/C189A^ did not profoundly promote the NHEJ efficiency (Figure 5f), indicating that both PHD domain and SDTE motif in PHRF1 are required to promote NHEJ repair.

### PHD domain and SDTE motif are essential for PHRF1 to DNA damage site

**Figure 6:**
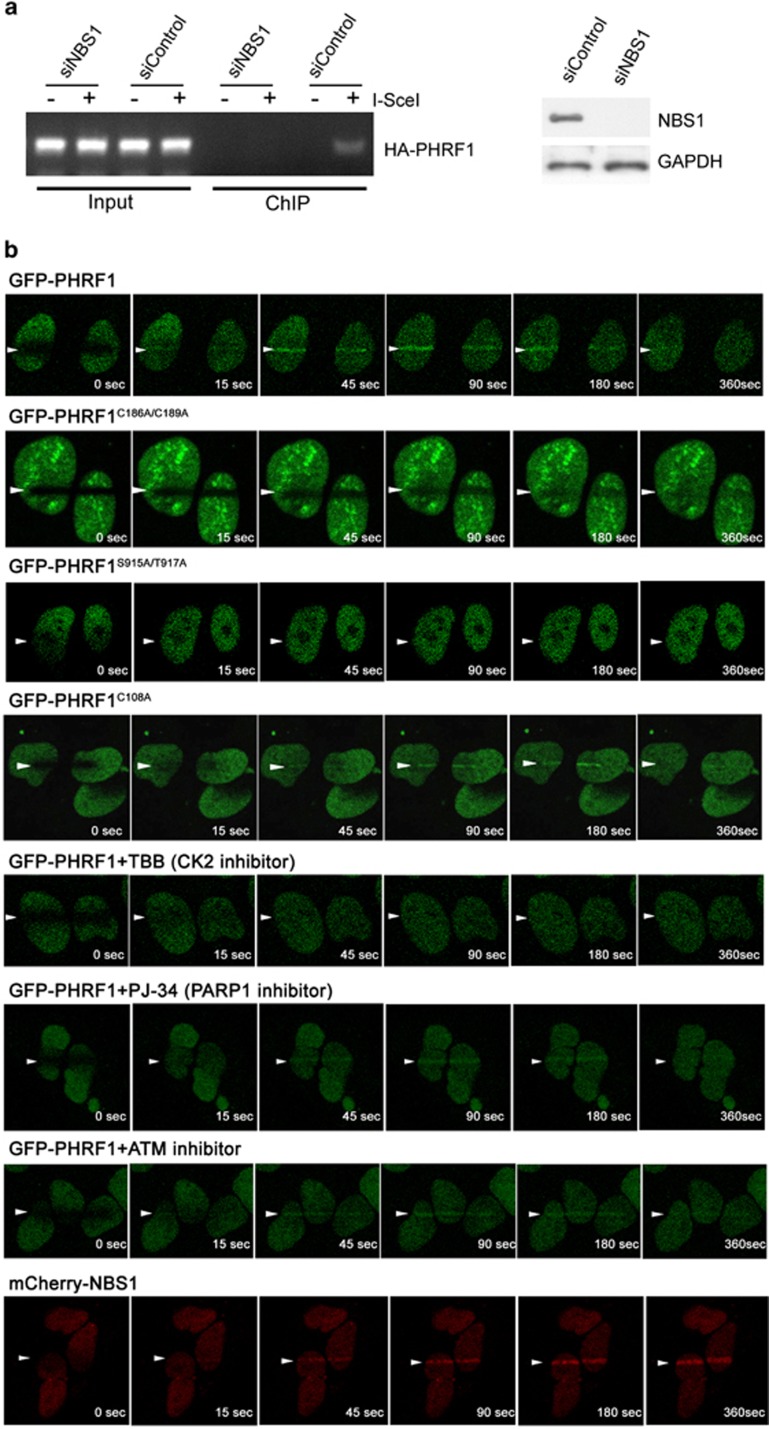
PHD domain and SDTE motif are required for PHRF1 to DNA damage sites. (**a**) NBS1 was depleted in NHEJ reporter H1299 cells and then I-SceI accompanied with empty vector or HA-PHRF1 were co-transfected into H1299 cells. ChIP was conducted using anti-HA antibody at 48 h after I-SceI transfection. (**b**) Live cell imaging shows the dynamic recruitments of GFP-PHRF1^C108A^, GFP-PHRF1^C186A/C189A^, GFP-PHRF1^S915A/T917A^, and GFP-PHRF1 in the presence of TBB (CK2 inhibitor), PJ-34 (PARP1 inhibitor), and ATM inhibitors to DNA damage sites generated by laser microirradiation in U2OS cells. mCherry-NBS1 was as a control. Note that PHRF1 and NBS1 almost simultaneously appeared at DNA damage sites before 180 s after laser irradiation. Arrows indicate the laser direction through the respective nuclei. GAPDH, glyceraldehyde 3-phosphate dehydrogenase; PARP1, poly(ADP-ribose) polymerase 1

To address whether PHRF1 was recruited to DSB sites with the assistance of NBS1, NBS1 was silenced in NHEJ reporter H1299 cells and DSBs were generated by I-SceI digestion. ChIP results showed that HA-PHRF1 was recruited to DNA damage sites only in the presence of NBS1 (Figure 6a). mCherry-NBS and GFP-PHRF1 almost simultaneously appeared at DNA lesions after laser microirradiation, but mCherry-NBS1 had a longer retention time at damaged sites (Figure 6b). To determine which region in PHRF1 is responsible for its recruitment to DNA damage sites, we monitored the recruitment of GFP-PHRF1 mutants to laser-induced DNA lesions. Interestingly, GFP-PHRF1 in the presence of ATM and PARP1 inhibitors and a dysfunctional E3 ligase mutant of PHRF1, GFP-PHRF1^C108A^, were able to appear at DNA damage sites. By contrast, GFP-PHRF1^C186A/C189A^ and GFP-PHRF1^S915A/T917A^ did not localize to laser-irradiated regions. Additionally, the addition of TBB, a CK2 inhibitor that potentially reduces the PHRF1^S915/T917^ phosphorylation, impaired the recruitment of PHRF1 to DNA damage sites, indicating that PHD domain and phosphorylatable SDTE motif are essential for localization of PHRF1 to DNA damage sites (Figure 6b).

### PHRF1 facilitates polyubiquitination of PARP1

**Figure 7:**
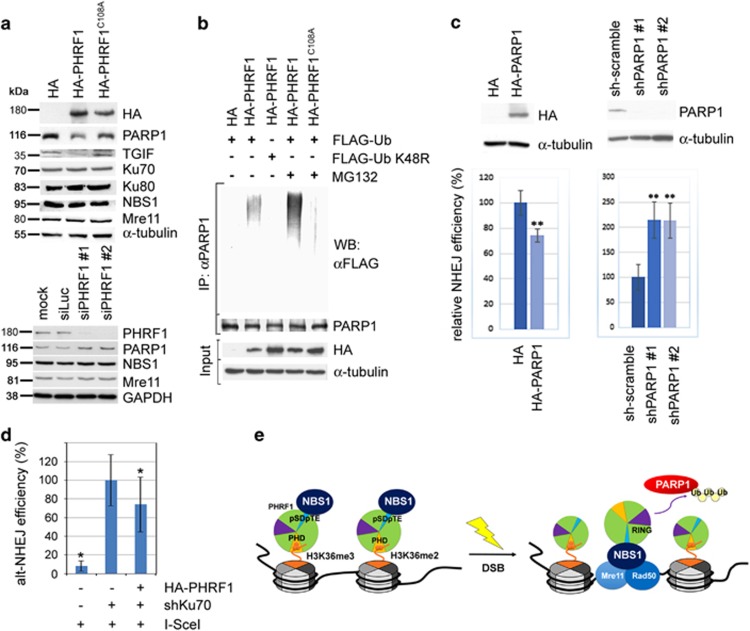
Poly(ADP-ribose) polymerase 1 (PARP1) is polyubiquitinated by PHRF1. (**a**) Total cell extracts harvested from HA-PHRF1/HA-PHRF1^C108A^-transfected HEK293T cells and siRNA-transfected HeLa cells were immunoblotted with indicated antibodies. (**b**) HA-PHRF1 and HA-PHRF1^C108A^ were co-transfected with FLAG-Ub or FLAG-Ub^K48R^ into HEK293T cells with the addition of MG132. Total cell extracts were immunoprecipitated (IP) with anti-PARP1 antibody and immunoblotted with anti-FLAG antibody to detect ubiquitinated PARP1. (**c**) NHEJ reporter H1299 cells were either transfected with HA-PARP1 or PARP1 short hairpin RNA (shRNA) for 24 h and then transfected with I-SceI expression plasmid for another 24 h. The proportion of enhanced green fluorescent protein (EGFP)-positive cells was determined by flow cytometry at 48 h after I-SceI transfection. Each experiment represented the mean±S.D. of three independent experiments. *P<0.05 and **P<0.01 compared with the control. (**d**) Ku70 were depleted by shRNA in alt-NHEJ reporter EJ2-GFP cells for 48 h and then co-transfected with I-SceI and HA-PHRF1 or empty HA vector for another 48 h. The proportion of GFP-positive cells was determined by flow cytometry. Each experiment represented the mean±S.D. of three independent experiments. *P<0.05 compared with the control. (**e**) A schematic model of PHRF1 in NHEJ repair. PHRF1 constitutively associates H3K36me2/3 via PHD domain and NBS1 by pSDpTE motif. When DSBs occur, PHRF1 may move to DSBs with the assistance of NBS1 to ubiquitinate PARP1 for NHEJ repair. GAPDH, glyceraldehyde 3-phosphate dehydrogenase; HA, hemagglutinin; Ub, ubiquitin; WB, western blot

PHRF1 contains a RING domain and this RING domain is responsible for its E3 ligase activity (Supplementary Figure S8). However, which proteins involved in NHEJ might be degraded by PHRF1 is unclear. Interestingly, the protein levels of Ku70, Ku80, NBS1, and Mre11 were mainly unchanged regardless of the expression levels of PHRF1. However, the amount of PARP1 is highly dependent on the presence of PHRF1, which was reduced in PHRF1-overexpressing HEK293T cells and increased in PHRF1-depleted HeLa cells (Figure 7a). The amount of PARP1 was restored in PHRF1^C108A^-transfected cells, and ubiquitin^K48R^ was not able to incorporate into PARP1 in the presence of PHRF1, suggesting that PARP1 is ubiquitinated for proteosomal degradation by PHRF1. Indeed, the ubiquitinated PARP1 was accumulated in the presence of a proteasome inhibitor MG132 (Figure 7b), indicating that the RING domain of PHRF1 is responsible for the ubiquitination of PARP1 *in vivo*. To investigate whether PARP1 affected NHEJ, the protein level of PARP1 was manipulated in H1299 NHEJ reporter cells. As anticipated, NHEJ capacity was significantly compromised, while the amount of PARP1 was increased and *vice versa* (Figure 7c). To measure alt-NHEJ efficiency, we depleted Ku70 in alt-NHEJ reporter EJ2-GFP U2OS cells^29^ and then co-transfected I-SceI and HA-PHRF1 into EJ2-GFP cells. Alt-NHEJ efficiency was reduced in the presence of PHRF1 compared with control HA vector (Figure 7d), indicating that PHRF1 may modulate alt-NHEJ by ubiquitinating PARP1.

## Discussion

Our results demonstrate the involvement of PHRF1 in NHEJ repair. First, the PHD domain of PHRF1 is responsible for its binding with H3K36me2 and H3K36me3. Second, the interaction of PHRF1 with NBS1 via SDTE motif is essential for the NHEJ activity. Third, PARP1 is identified as one of the ubiquitination targets of PHRF1. Finally, on the basis of our findings, we conclude that PHRF1 may move to DSBs with the assistance of H3K36me2/me3 and NBS1 and then ubiquitinate PARP1 to trigger subsequent NHEJ (Figure 7e). However, the colocalization of PHRF1 with H3K36me2/3 and NBS1 is not affected in *ATM*-deficient cells (Supplementary Figure S9) and PHRF1^S925A/S1389A^ did not reduce the NHEJ efficiency in NHEJ reporter H1299 cells (Figure 5f), suggesting that ATM is not required for the interaction of PHRF1 with H3K36me2/3 and NBS1.

Dimethylation of H4K20me2 by histone methyltransferase MMSET is critical for the recruitment of 53BP1 to facilitate NHEJ. However, several studies fortify alternative pathways for 53BP1 recruitment to DNA damage foci. H4K16 acetylation antagonizes 53BP1 binding to H4K20me2, while H4K16 deacetylation facilitates 53BP1 foci formation and NHEJ.^30^ Additionally, TIP60-dependent H4 acetylation can diminish 53BP1 binding to H4K20me2 through the disruption of a bridge between H4K16 and Glu1551 in 53BP1.^31^ Although H3K36me2 near the DNA damage sites enhances the association of Ku70 and NBS1 to promote NHEJ repair,^20^ it is unclear whether H3K36 methylation has a direct impact for H4K20me2 to recruit 53BP1. As we did not find PHRF1 colocalization with 53BP1 and there was no significant change for 53BP1 foci formation in PHRF1-depleted and -overexpressing cells (Supplementary Figure S3), PHRF1 may not be an alternative option to link H3K36me2/3 for 53BP1 recruitment and modulate NHEJ repair.

Methylation of H3K36 is generally associated with ‘open' euchromatin and permissible for RNA pol II to activate gene transcription, but H3K36 methylation is also involved in alternative splicing, transcriptional repression, DNA repair, and recombination.^25^ Rat PHRF1 interacts with the C-terminal domain of RNA polymerase II revealed by yeast two-hybrid assay and immunoprecipitation analysis.^32^ Although PHRF1 preferentially associates with H3K36me3 in active euchromatin and possibly participates in gene transcription, it is feasible that PHRF1 may assist DNA repair in euchromatin. Recently, Li *et al.*^33^ identified a novel role of H3K36me3 to facilitate DNA mismatch repair (MMR) by targeting the MMR machinery to chromatin.^33^ It has been described that MMR proteins MLH1, EXO1 and MSH2 are important for class-switch recombination (CSR) and are capable of converting DNA nicks and point mutations into dsDNA breaks for both C-NHEJ and alt-NHEJ pathways of CSR.^34^ Genetic screening of systemic sclerosis (SSc)- and systemic lupus erythematosus (SLE)-related chronic autoimmune diseases showed high frequencies of single-nucleotide polymorphisms (SNP) in IRF7/PHRF1 locus.^35^ Although the functional relationship between PHRF1 and SSc/SLE is unclear, it is plausible to speculate that PHRF1 may participate in V(D)J recombination or CSR, both need NHEJ to facilitate antibody production.

Our study also raises interesting questions regarding the interaction of PHRF1 with NBS1 and the ubiquitination of PARP1. MDC1 contains six SDTD clusters (a.a. 210–460) to interact with NBS1.^26, 27, 28^ By contrast, PHRF1 contains only one SDTE motif (a.a. 915–918). Substitution of this SDTE to ADAE in HA-PHRF1 greatly decreased its interaction with NBS1 and further impaired NHEJ efficiency (Figure 5). It would be interesting to determine under which condition or which factor directs NBS1 to choose among MDC1 and PHRF1. PARP1 is suggested to be involved in the activation of alt-NHEJ pathway^7^ and has been described to be ubiquitinated by CHFR and Iduna.^36, 37, 38^ CHFR mediates polyubiquitination/degradation of PARP1 and protects cells against DNA damage insults. Iduna is a poly(ADP-ribose)-dependent E3 ubiquitin ligase that promotes PARP1 ubiquitination and DNA repair. Is PHRF1 a redundant E3 ligase to promote PARP1 degradation or is it required for PARP1 ubiquitination under certain circumstances? We preferentially favor the notion that the recruitment of PHRF1 to DSBs is a critical phase to ubiquitinate PARP1 for NHEJ. If it is so, PHRF1 might be an alternative option by choosing the appropriate DSB repair to maintain genome integrity.

## Materials and Methods

### Cells lines

HEK293T cells were maintained in DMEM medium with 10% FBS. H1299dA3-1 human lung cancer cells harboring an integrated pIRES-TK-EGFP NHEJ reporter was a gift from Dr. T Kohno^25^ and EJ2-GFP U2OS cells was a gift from Dr. JM Stark^29^. U2OS cells containing DR-GFP HR reporter were cultured in McCoy's 5a medium supplemented with 10% FBS.

### Antibodies

Mouse anti-PHRF1 monoclonal antibody was raised against 6xHis-tagged PHRF1 recombinant protein (a.a.1298–1649). Mouse anti-HA were purchased from Santa Cruz Biotech (Santa Cruz, CA, USA). Rabbit anti-methylated histone antibodies were obtained from Abcam (Cambridge, UK). Mouse anti-α-tubulin antibody was from Sigma-Aldrich (St. Louis, MI, USA). Rabbit anti-NBS1, Ku70 and Ku80 antibodies were from GeneTex (Irvine, CA, USA). Rabbit anti-PARP1 antibody was from Cell Signaling (Irvine, CA, USA).

### Small interfering RNA

The small interfering RNA (siRNA) siPHRF1 no. 1, 5′-GGACAAAGUGUUAGAGGUAdTdT-3′ and siPHRF1 no. 2, 5′-CGGAAGAGCUCUAUGGGAAdTdT-3′ were synthesized by GE Healthcare Dharmacon (Lafayette, CO, USA) to knockdown the expression of PHRF1.

### Whole-cell extracts, nuclear extracts, and chromatin isolation

Whole-cell extracts were prepared with RIPA lysis buffer. To prepare cytosolic and nuclear extracts, 1 × 10^8^ cells were lysed in buffer A (10 mM HEPES (pH 7.9), 10 mM KCl, 1.5 mM MgCl_2_, 0.34 M sucrose, 10% glycerol, 1 mM DTT, 0.1% Triton X-100, protease inhibitors). After incubation at 37 °C for 5 min, nuclei were lysed in 1 ml of buffer B (3 mM EDTA, 0.2 mM EGTA, 1 mM DTT, protease inhibitors). Insoluble chromatin was separated via centrifugation (5 min, 2,500 r.p.m.) and resuspended in 0.5 ml Laemmli buffer and then sonicated for 5 min for SDS-PAGE.

### Lentivirus production

The packaging vector pCMVdeltaR8.91, the VSV-G envelope glycoprotein vector pMD.G, and the full-length of PHRF1 in the pLenti6/V5 vector were co-transfected into HEK293 T cells. After 48 h incubation, the viral particles were collected to infect U2OS cells in the presence of polybrene (8 *μ*g/ml) for viral infection. Stable clones were selected with blasticidine (Thermo Scientific, Waltham, MA, USA) for 21 days.

### Laser microirradiation

Cells were presensitized with 10 *μ*M of 5-bromo-2-deoxyuridine (BrdU; Sigma-Aldrich) for 24 h at 37 °C. Laser microirradiation was carried out with a Leica SP5X inverted confocal microscope (Leica Microsystems, Buffalo Grove, IL, USA) and a 405 nm laser diode with full output settings. Two scans were used to generate DNA damage restricted to the laser path with minimal cellular toxicity. Cells were either left untreated or in the presence of TBB (75 *μ*M; Sigma-Aldrich) for 6 h, PJ-34 (10 *μ*M; Sigma-Aldrich) for 2 h, or ATM inhibitor (10 *μ*M; Calbiochem, Billerica, MA, USA) for 2 h.

### Alkali comet assay

Single-cell gel electrophoretic comet assays were performed under alkaline conditions using Trevigen CometAssay Kit (Gaithersburg, MD, USA). Briefly, 2 × 10^5^ cells were exposed to etoposide (20 *μ*M) for 30 min in HeLa cells and 1 h in U2OS cells, respectively. Cells were gently mixed with 1% low melting agarose at 37 °C at the ratio of 1 : 10 (v/v) and then spread onto slides. The slides were immersed in the alkali unwinding solution (0.3 M NaOH, 1 mM EDTA, pH>13) for 1 h at 4 °C, subjected to electrophoresis at 20 V for 30 min, and stained in DAPI (4',6-diamidino-2-phenylindole; 10 mg/ml) for 15 min. All images were recorded using a Leica fluorescence microscope.

### Colony formation assay

Five thousand cells plated in 6-well plates were exposed to CPT (2 *μ*M) for 24 h or etoposide (10 *μ*M) for 1 h in PHRF1-depleted cells and CPT (5 *μ*M) for 24 h or etoposide (20 *μ*M) for 1 h in PHRF1-overexpressing cells, respectively. After incubation for 10–12 days, the surviving colonies were stained with 0.25% crystal violet in ddH_2_O. The stained colonies with diameter >0.1 mm were calculated.

### Peptide synthesis and *in vitro* pull-down assay

Biotin-labeled histone peptides (H3K36, APATGGVKKPHRYRP; H3K36me1, APATGGVK^me1^KPHRYRP; H3K36me2, APATGGVK^me2^KPHRYRP; H3K36me3, APATGGVK^me3^KPHRYRP; H4K20me2, GGAKRHRK^me2^VLRDNIQ; H4K20me3, GGAKRHRK^me3^VLRDNIQ were synthesized by Genemed Synthesis (San Antonio, TX, USA). Biotin-labeled NBS1 peptides, SDTE (AVAAEGASDTEREEPTE; a.a. 908–924), ADAE (AVAAEGAADAEREEPTE), pSDTE (AVAAEGApSDTEREEPTE), SDpTE (AVAAEGASDpTEREEPTE), and pSDpTE (AVAAEGApSDpTEREEPTE), were synthesized at the Institute of Biological Chemistry at Academia Sinica (Taipei, Taiwan). Five hundred micrograms of HeLa whole-cell extracts were incubated with biotin-labeled peptides at 4 °C for 3 h, and the complex was then incubated with streptavidin beads (Sigma-Aldrich) for another 3 h. The immunoprecipitated complex were resolved by SDS-PAGE and analyzed by immunoblotting.

### *In vivo* end-joining assay

Linearized plasmid pGL32 (Promega, Madison, WI, USA) by *Hin*dIII was purified with phenol/chloroform extraction and ethanol precipitation, and then transfected into HEK293T cells. At 48 h posttransfection, luciferase activity was assayed using Bright-Glo Luciferase Assay Kit (Promega). The transfection of SV40-*β*-gal plasmid was used as the normalized control.

### *In vivo* ubiquitination

HEK293T cells were co-transfected with FLAG-tagged ubiquitin and HA-tagged PHRF1^WT^ or PHRF1 RING mutants (HA-PHRF1^C108A^ and HA-PHRF1^I110S/W137R^) with or without the addition of MG132 proteasome inhibitor. Total cell extracts from transfected cells were harvested in RIPA buffer at 40 h after transfection for immunoblotting analysis.

## Abbreviations

Alt-NHEJ: alternative non-homologous end-joining
ATM: ataxia telangiectasia-mutated
ATR: ataxia telangiectasia and Rad3-related
BrdU: 5-bromo-2-deoxyuridine
ChIP: chromatin immunoprecipitation
CPT: camptothecin
CSR: class-switch recombination
DSB: double-strand break
H3K36: histone H3 lysine 36
H3k36me2: dimethylated H3K36
H3K36me3: trimethylated H3K36
H4K20me2: dimethylated H4K20
HR: homologous recombination
MMR: DNA mismatch repair
MRN: Mre11-RAD50-NBS1
NHEJ: non-homologous end-joining
PHD: plant homeodomain
Rif1: Rap1-interacting factor 1
SLE: systemic lupus erythematosus
SNP: single-nucleotide polymorphisms
SSc: systemic sclerosis
Ub: ubiquitin
