# Supplementary material for: PHRF1 promotes genome integrity by modulating non-homologous end-joining
Source: Cell Death Dis. 2015 Apr 9;6(4):e1716–. doi: 10.1038/cddis.2015.81 (PMC4650548; doi:10.1038/cddis.2015.81)
Supplement: Supplementary Data [file cddis201581x1.pdf]

Supplementary Data

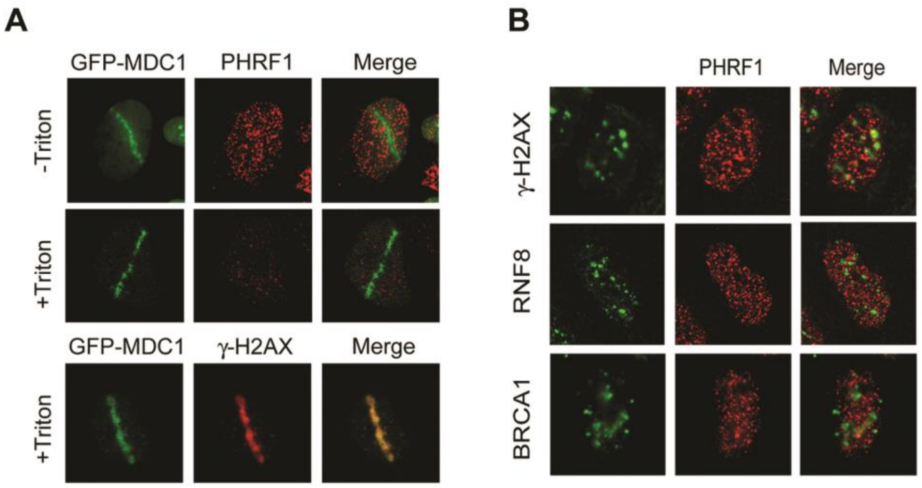

Supplementary Figure S1. PHRF1 did not localize to γ-H2AX associated nuclear foci. (A) Stably expressing GFP-MDC1 U2OS cells were microirradiated with laser light (405 nm), 0.5% Triton-extracted, and then immunostained at 2 h post laser irradiation with indicated antibodies. (B) HeLa cells were treated with CPT (10 μM) for 3 h and fixed with 4% paraformaldehyde for immunofluorescence staining. Anti-PHRF1 antibody was simultaneously incubated with anti-γ-H2AX, RNF8, and BRCA1 antibodies, respectively.

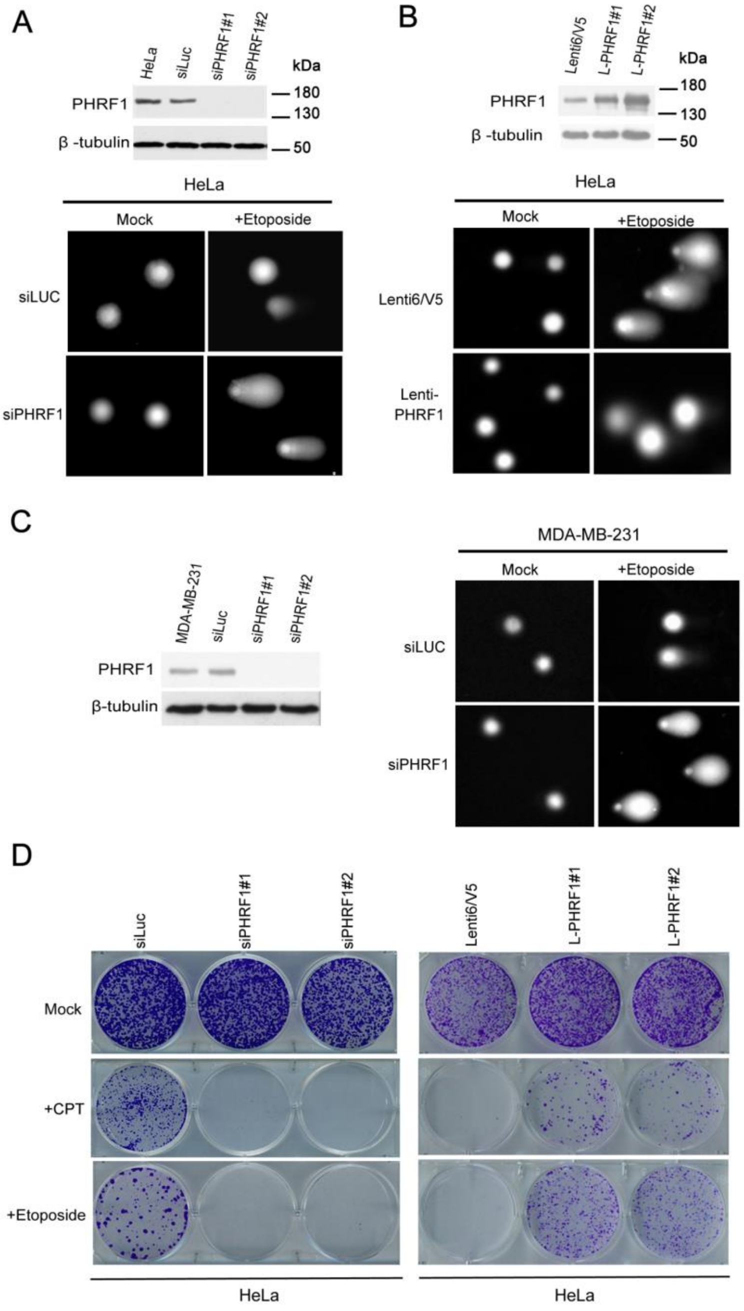

Supplementary Figure S2. Comet assay and clonogenic assay in HeLa cells and MDA-MB-231 cells. (A) PHRF1 depleted HeLa cells were exposed to etoposide for 30 min for the comet assay. (B) Control and PHRF1 overexpressing HeLa cells were exposed to etoposide for 1 h for the comet assay. (C) PHRF1 depleted breast cancer MDA-MB-231 cells were exposed to etoposide for 30 min for the comet assay. (D) For clonogenic assays, PHRF1-depleted HeLa cells were exposed to CPT (2 μM) for 24 h or etoposide (10 μM) for 1 h and PHRF1-overexpressing cells were treated with CPT (5 μM) for 24 h or etoposide (25 μM) for 1 h. Cells were allowed to grow for 10 days until colonies were formed.

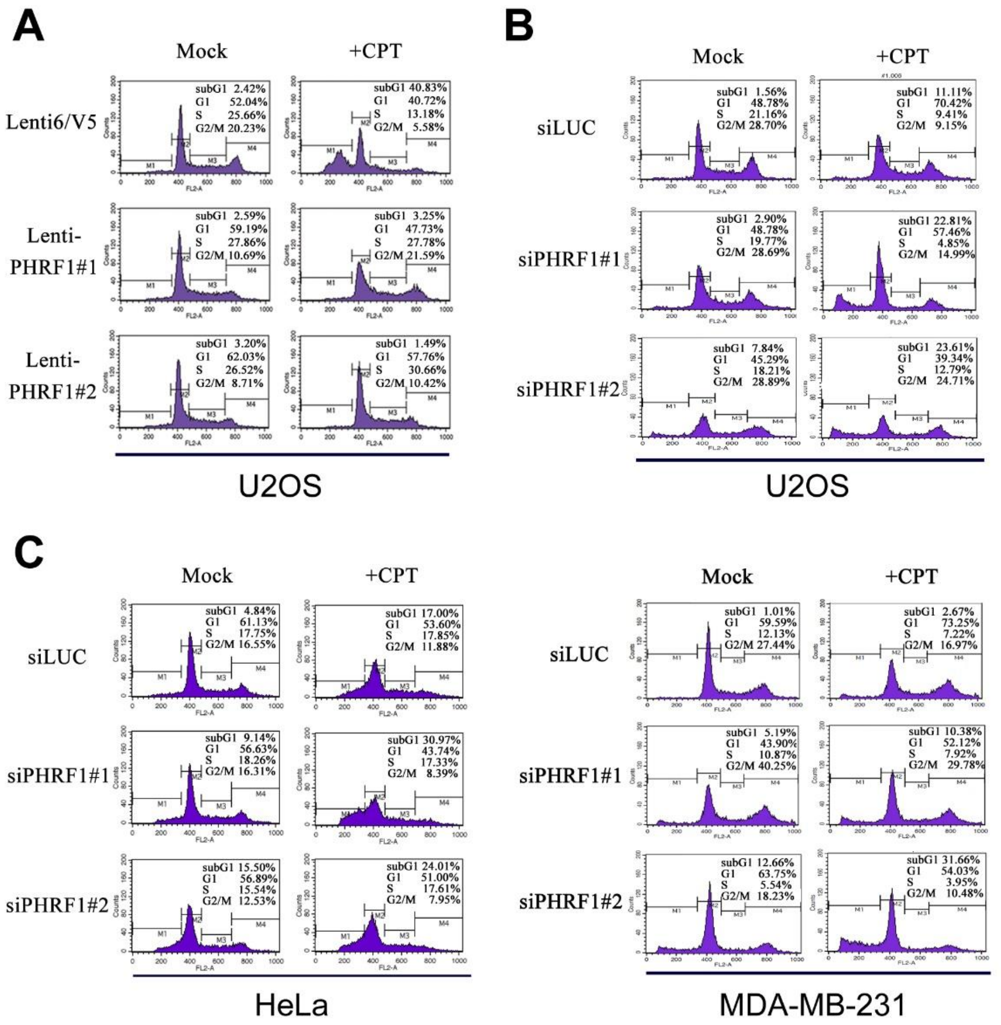

Supplementary Figure S3. The effects of overexpression and knockdown of PHRF1 on cell cycle. (A) DNA contents were determined by flow cytometry before and after CPT exposure (5 μM) for 24 h in PHRF1 overexpressing U2OS cells. (B) DNA contents were determined by flow cytometry before and after CPT exposure (5 μM) for 24 h in PHRF1 knockdown U2OS cells. (C) DNA contents were determined by flow cytometry before and after CPT exposure (5 μM) for 24 h in PHRF1-depleted HeLa and MDA-MB-231 cells. Note that the increased sub-G1 population in PHRF1 knockdown cells but decreased in PHRF1 overexpressing cells in response to CPT treatment.

.

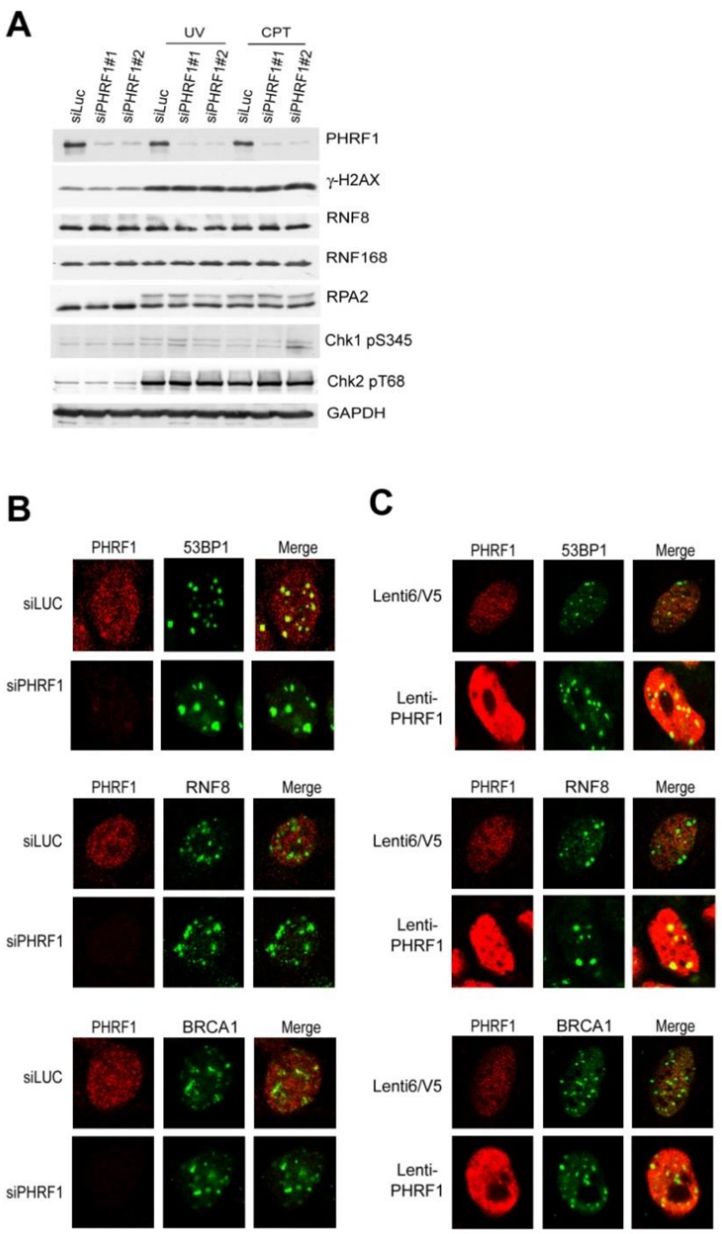

Supplementary Figure S4. PHRF1 did not affect DNA damage response. (A) HeLa cells were transfected with control or PHRF1 siRNAs. Seventy-two hours after siRNA transfection, cells were treated with CPT (10 μM) for 3 h and whole cell extracts were prepared for immunoblotting analysis using indicated antibodies. (B) HeLa cells were transfected with control or PHRF1 siRNAs. Seventy-two hours after siRNA transfection, cells were treated with CPT for 3 h and then simultaneously immunostained with anti-PHRF1 and indicated antibodies. (C) Control and PHRF1 overexpressing U2OS cells were exposed to CPT (10 μM) for 3 h and then simultaneously immunostained with anti-PHRF1 and indicated antibodies. Note that the foci formation of RNF8, 53BP1 and BRCA1 were not affected in PHRF1-depleted and overexpressing cells.

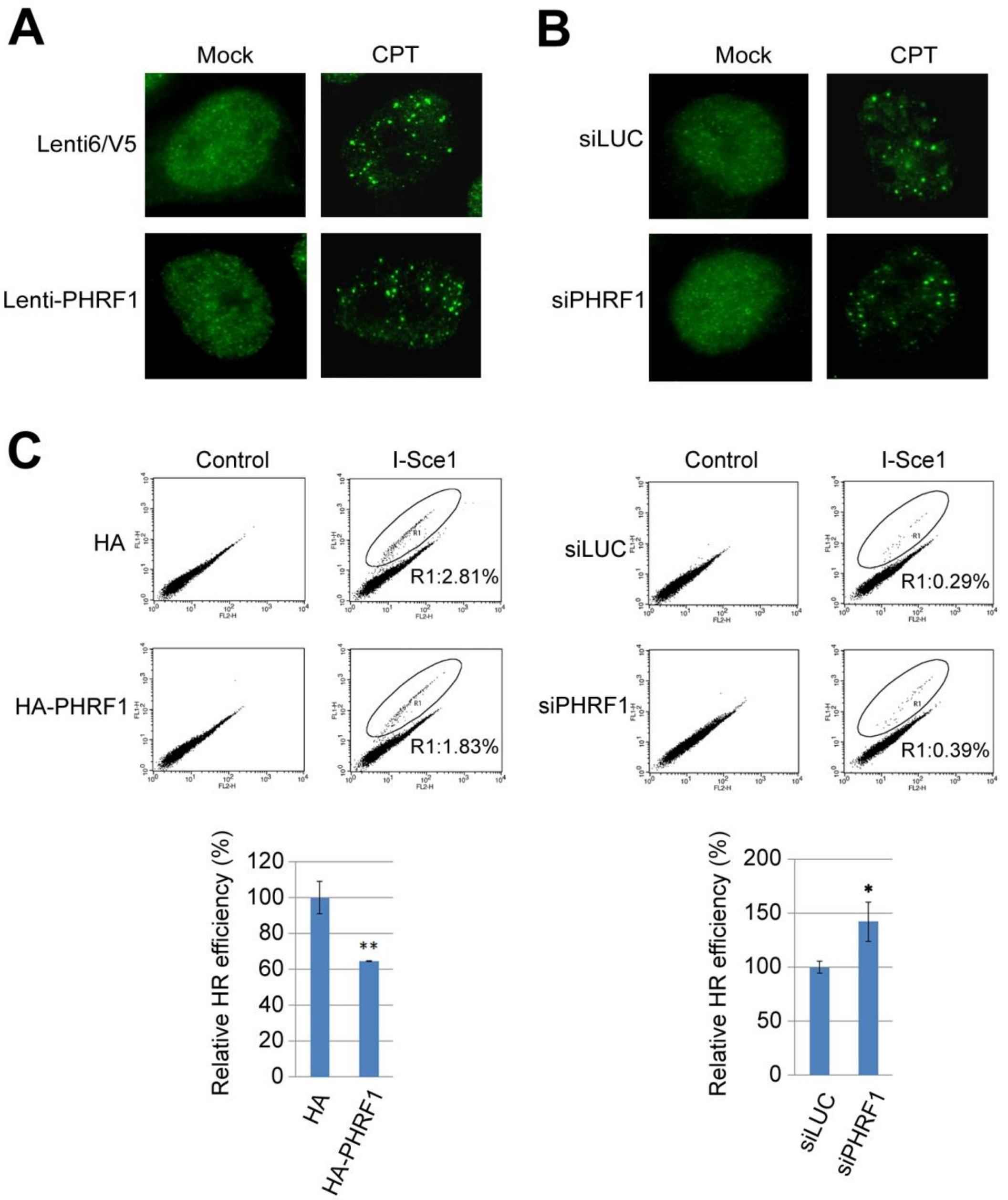

Supplementary Figure S5. PHRF1 was not required for Rad51 foci formation but reduced homologous recombination. (A) Control and PHRF1 overexpressing U2OS cells were exposed to CPT for 3 h. Cells were fixed in 4% paraformaldehyde and immunostained with anti-Rad51 polyclonal antibody. (B) HeLa cells were transfected with control and PHRF1 siRNAs for 72 h and then exposed to CPT for 3 h. Cells were fixed in 4% paraformaldehyde and immunostained with anti-Rad51 polyclonal antibody. (C) DR-GFP HR reporter U2OS cells were transfected with HA-PHRF1 and PHRF1 siRNAs, respectively. The expression of green fluorescence protein was measured by flow cytometry. Each experiment represented the means±standard deviations of three independent experiments.

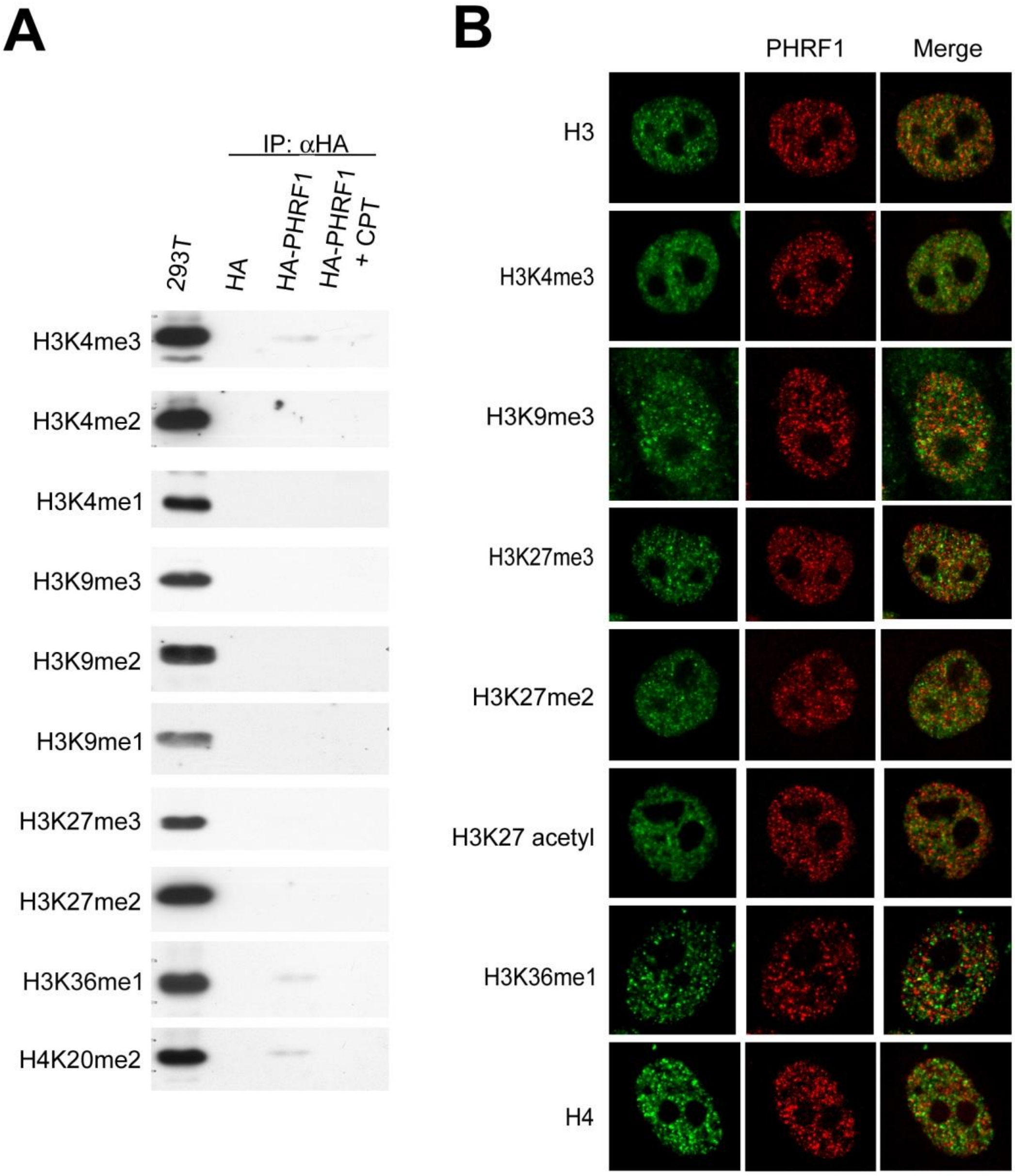

Supplementary Figure S6. PHRF1 did not associate with methylated H3K4, H3K9, H3K27, and H4K20 histones. (A) Empty HA vector and HA-PHRF1 were transfected into HEK293T cells and cell extracts were immunoprecipitated with anti-HA agarose. Immunoblotting analysis was conducted using indicated anti-methyl histone antibodies. (B) HeLa cells were fixed in 4% paraformaldehyde and simultaneously labeled with a mixture of anti-PHRF1 mAb (red) and indicated anti-methyl histone antibodies (green). A few amounts of H3K4me3, H3K36me1, and H4K20me2 were immunoprecipitated by HA-PARP1, but very little colocalization of these methylated histones with PHRF1.

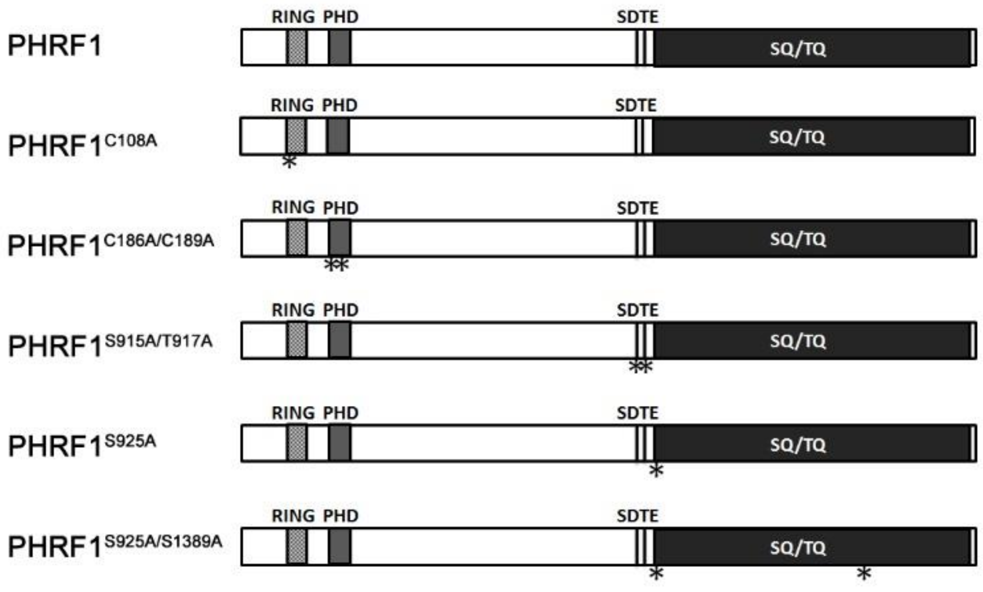

Supplementary Figure S7. Schematic representation of PHRF1's substitution mutants. * indicates the position of substituted protein residues. Note that there are seven SQ/TQ motifs in PHRF1.

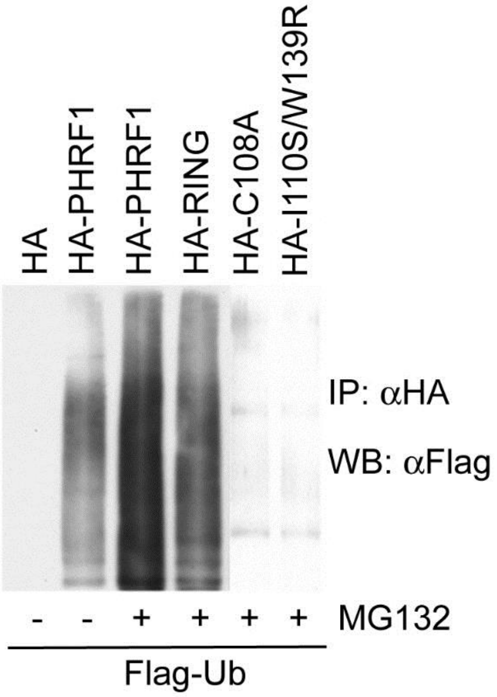

Supplementary Figure S8. PHRF1 is polyubiquitinated. HA vector, HA-PHRF1, HA-RING domain, and two PHRF1 E3 ligase mutants, HA-PHRF1^{C108A} (HA-C108A) and HA-PHRF1$^{I110S/W139R}$ (HA-I110S/W139R) were co-transfected with Flag-Ubiquitin into HEK293 cells with or without the addition of MG132. Total cell extracts were immunoprecipitated with anti-HA agarose and immunoblotted with anti-Flag antibody.

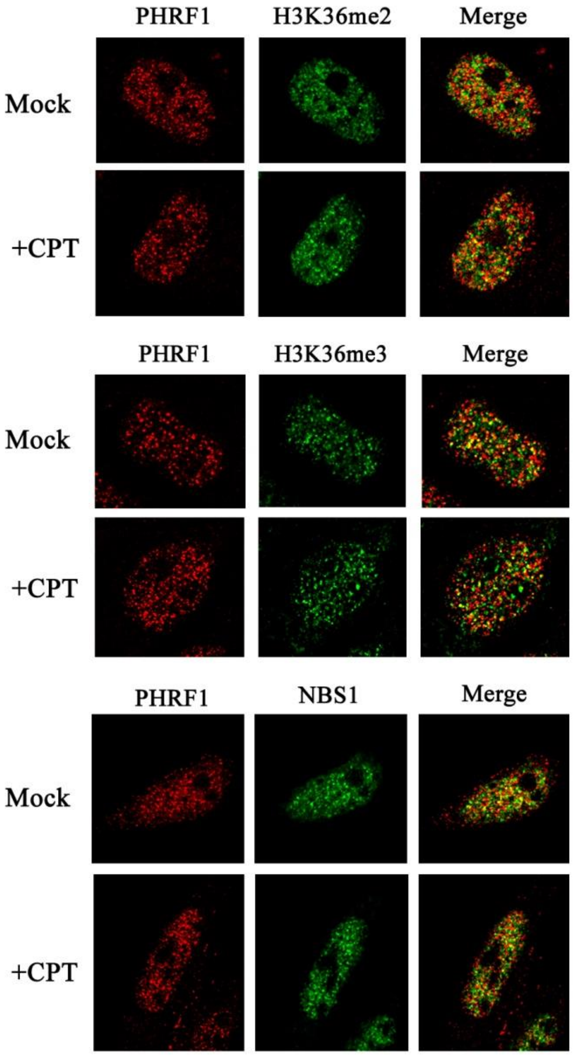

Supplementary Figure S9. Human SV-40-immortalized *ATM* fibroblasts from Coriell Cell Repositories were exposed to CPT and then fixed in 4% paraformaldehyde and simultaneously labeled with a mixture of anti-PHRF1 mAb (red) and indicated antibodies (green).

Supplementary Materials and Methods

Antibodies. Mouse anti-RPA2, anti-Chk1pS345, anti-Chk2pT68, and anti-γ-H2AX were from Cell Signaling (Danvers, MA). Rabbit anti-RNF8, anti-RNF168, and anti-MSH6 antibodies were from Abcam (Cambridge, UK). Mouse anti-GAPDH antibody was from Novus Biologicals (Littleton, CO). Mouse anti-α-tubulin antibody was from Sigma-Aldrich (St. Louis, MI). Rabbit anti-NBS, Mre11, Ku70, Ku80, and BRCA1 antibodies were from GeneTex (Irvine, CA).

Homologous recombination assay. U2OS cells with the integrated homologous recombination reporter DR-GFP was a gift from Dr. Jeremy Stark (City of Hope, USA). One day after transfection with siRNA, U2OS-DR-GFP cells were co-transfected with an I-SceI expression vector (pCBA-I-SceI). Flow cytometry was conducted to measure the expression of GFP protein.

Laser microirradiation. Cells were pre-sensitized with 10 μM of 5-bromo-2.-deoxyuridine (BrdU; Sigma Aldrich) in normal medium for 24 h at 37℃. Laser micro-irradiation was carried out with a Leica SP5 X Inverted Confocal Microscope and a 405 nm laser diode. Laser settings (full output, 2 scans) were used to generate DNA damage that was restricted to the laser path in a pre-sensitization-dependent manner with minimal cellular toxicity.
